# From Compressed-Sensing to Artificial Intelligence-Based Cardiac MRI Reconstruction

**DOI:** 10.3389/fcvm.2020.00017

**Published:** 2020-02-25

**Authors:** Aurélien Bustin, Niccolo Fuin, René M. Botnar, Claudia Prieto

**Affiliations:** ^1^Department of Biomedical Engineering, School of Biomedical Engineering and Imaging Sciences, King's College London, London, United Kingdom; ^2^Escuela de Ingeniería, Pontificia Universidad Católica de Chile, Santiago, Chile

**Keywords:** cardiac MRI, AI, reconstruction, dictionary learning, deep learning, undersampling

## Abstract

Cardiac magnetic resonance (CMR) imaging is an important tool for the non-invasive assessment of cardiovascular disease. However, CMR suffers from long acquisition times due to the need of obtaining images with high temporal and spatial resolution, different contrasts, and/or whole-heart coverage. In addition, both cardiac and respiratory-induced motion of the heart during the acquisition need to be accounted for, further increasing the scan time. Several undersampling reconstruction techniques have been proposed during the last decades to speed up CMR acquisition. These techniques rely on acquiring less data than needed and estimating the non-acquired data exploiting some sort of prior information. Parallel imaging and compressed sensing undersampling reconstruction techniques have revolutionized the field, enabling 2- to 3-fold scan time accelerations to become standard in clinical practice. Recent scientific advances in CMR reconstruction hinge on the thriving field of artificial intelligence. Machine learning reconstruction approaches have been recently proposed to learn the non-linear optimization process employed in CMR reconstruction. Unlike analytical methods for which the reconstruction problem is explicitly defined into the optimization process, machine learning techniques make use of large data sets to learn the key reconstruction parameters and priors. In particular, deep learning techniques promise to use deep neural networks (DNN) to learn the reconstruction process from existing datasets in advance, providing a fast and efficient reconstruction that can be applied to all newly acquired data. However, before machine learning and DNN can realize their full potentials and enter widespread clinical routine for CMR image reconstruction, there are several technical hurdles that need to be addressed. In this article, we provide an overview of the recent developments in the area of artificial intelligence for CMR image reconstruction. The underlying assumptions of established techniques such as compressed sensing and low-rank reconstruction are briefly summarized, while a greater focus is given to recent advances in dictionary learning and deep learning based CMR reconstruction. In particular, approaches that exploit neural networks as implicit or explicit priors are discussed for 2D dynamic cardiac imaging and 3D whole-heart CMR imaging. Current limitations, challenges, and potential future directions of these techniques are also discussed.

## Introduction

Magnetic resonance imaging (MRI) is a valuable tool for the non-invasive assessment of cardiovascular disease. Cardiac MR (CMR) imaging has been established as a clinically important technique for the assessment of cardiac morphology, function, perfusion, viability, and more recently quantitative myocardial tissue characterization (1–3). CMR is currently used to diagnose congenital heart disease (CHD), ischemic heart disease, valvular heart disease, pericardial lesions, cardiac tumors and cardiomyopathies, among others (4, 5). However, CMR suffers from long acquisition times due to the need of obtaining images with high temporal and spatial resolution, different contrasts, and/or whole-heart coverage. In addition, both cardiac and respiratory-induced motion of the heart during the acquisition need to be accounted for, further increasing the scan time.

Several technical advances have been proposed during the last decades to improve CMR, including the development of efficient pulse sequences to speed up the scan and improve the contrast of the images, the development of motion compensation techniques to account for the respiratory and cardiac induced movement of the heart, the use of multiple radio-frequency receiver coils for parallel imaging (PI), and the development of undersampled reconstruction techniques to acquire less data than needed (in the Nyquist sense) and thus accelerate the acquisition. PI allows to decrease the scan time by reducing the number of phase increment steps (undersampling) and exploiting the sensitivity encoding of the multiple receiver coils to recover the non-acquired data. PI has been widely integrated into commercial MR systems and is routinely used in clinical practice. Undersampled reconstruction techniques such as compressed sensing (CS) have been also employed to accelerate CMR imaging. CS works under the assumption that the k-space data is randomly undersampled, the image has a sparse representation in some pre-defined basis or dictionary, and a non-linear reconstruction is performed to enforce the sparsity of the image and consistency with the acquired MR data. In practice, CS-based reconstruction techniques employ pseudo-random trajectories (usually with variable density) along with one or several (e.g., spatial and temporal dimensions) sparse transforms such as finite differences (e.g., total variation) or wavelets operators. Early 2017, the U.S. Food and Drug administration (FDA) cleared the CS technology to enable the fast acquisition of CMR images, thus officially opening the door to the broader clinical use of this technique (6–8).

Recent efforts have been made to further improve CS-based reconstruction quality by learning dictionary-based representations of the sparse domain from the acquired data itself (or jointly during reconstruction) instead of exploiting known analytical transform domains. However, CS-based reconstruction techniques usually suffer from long computational times and their performance depends on the choice of the sparsity representation and the tuning of the corresponding reconstruction parameters. More recently deep neural networks (DNN) have been proposed to overcome these challenges by learning optimal reconstruction parameters and/or transforms from the data itself and enabling extremely fast computational times (after training), promising to further advance the field of CMR reconstruction.

In this review paper, we first briefly discuss the CS and dictionary learning models, which offer a framework for sparse signal recovery and low-dimensional signal models and serve as a background for the following section. Recent representative advances in deep learning (DL) for CMR reconstruction are next discussed, highlighting theoretical developments and cardiac applications.

## Transform and Data-Driven CMR Reconstruction

This section briefly introduces the key concepts that underlie MR image reconstruction as an inverse problem, that will serve as background material to the rest of the review. CS-based and dictionary learning models for CMR reconstruction are also discussed. We refer the reader to Ye (9) and Jaspan et al. (10) for further discussion on the application of CS to MR image reconstruction.

### MR Reconstruction as an Inverse Problem

The general (discretized) principles of MR signal generation and image formation can be expressed as a system of linear equations (11):

(1) $$\begin{array}{l} s=E\rho \end{array}$$

**Figure 1:**
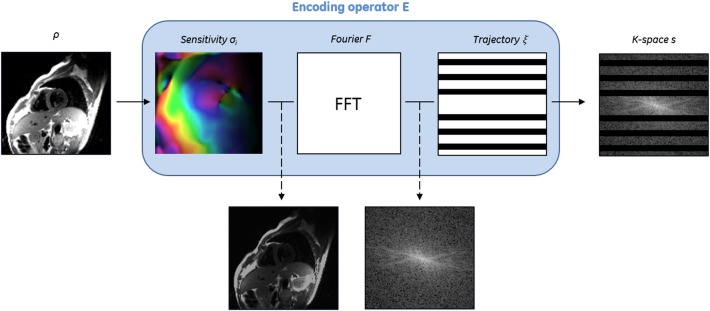
Description of the encoding operator *E* for CMR reconstruction.

Where the MR encoding operator *E* includes the coil sensitivity profiles, the Fourier transform and the sampling mask, ρ is the image to be recovered and *s* is the acquired k-space data (Figure 1). The image ρ is thus reconstructed by solving an inverse problem that aims to recover an estimate of ρ from the *known* encoding operator *E* and the acquired signal *s*. This inverse problem is ill-posed, i.e., not all the following well-posedness conditions are satisfied: (i) existence of the solution, (ii) uniqueness of the solution, and (iii) stability of the solution (i.e., small disturbances in *s* do not lead to large perturbations in ρ). The main factors that make MR reconstruction an ill-posed problem include the large scale of the optimization, the system imperfections (e.g., coils sensitivities, signal model simplifications), the limited amount of phase increment steps (undersampling) and the acquisition noise which corrupts the signal.

To overcome the ill-posed nature of the MR image reconstruction problem, this is typically reformulated as a regularized optimization:

(2) $$\begin{array}{l} \hat{\rho }={\text{argmin}}_{\rho }\;\|E\rho -s{\|}_{2}^{2}+\lambda R(\rho ) \end{array}$$

where the image $\hat{\rho }$ is recovered by balancing between a regularization term *R*(ρ), which is added as an additional constraint to stabilize the solution, and a data consistency ${\left\|E\rho -s\right\|}_{2}^{2}<\epsilon$, where ϵ is the noise level. The weighting parameter λ controls the degree of regularization and needs to be chosen according to the noise level of the acquired data. Especially, considering sparsity priors and statistical properties of the MR images to regularize the reconstruction problem have shown great promise. The application of these techniques to speed CMR imaging is the topic of the following subsections.

### CS for CMR Imaging

CS MRI reconstruction assumes that the k-space data is pseudo-randomly undersampled, the image admits a sparse representation in some transform domain Φ, and a non-linear reconstruction is performed to enforce data consistency and sparsity of the MR image in the transform domain. A natural approach to enforce sparsity is by replacing the regularization term in Equation (2) by the *l*_0_ (pseudonorm) of the sparse coefficients (12), which counts the number of non-zero entries. However, since the *l*_0_ “norm” does not satisfy the convexity property of a norm and leads to an NP-hard combinatorial problem, approximate solutions are considered instead by replacing the *l*_0_ term by the convex *l*_1_-norm (13):

(3) $$\begin{array}{l} \hat{\rho }={\mathrm{argmin}}_{\rho }\|E\rho -s{\|}_{2}^{2}+\lambda \|\Phi \rho {\|}_{1} \end{array}$$

**Figure 2:**
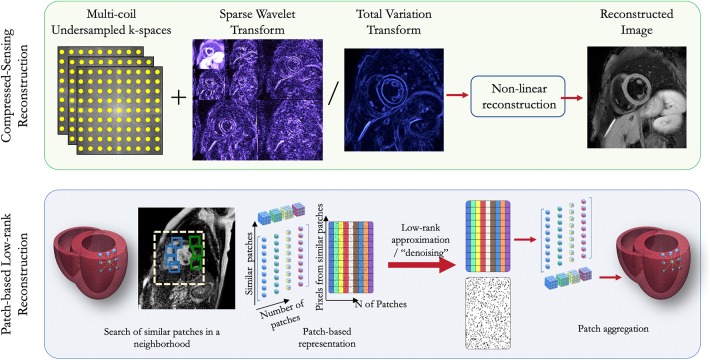
Schematic diagram of compressed sensing and patch-based low-rank reconstructions for CMR.

The problem in Equation (3) is convex and can be solved with a variety of regularization and convex optimization techniques. In cardiac MRI, Φ can be chosen e.g., as the temporal Fourier transform, spatio-temporal total variation, or spatio-temporal wavelets (Figure 2). CS has been extensively used in numerous cardiac applications, such as cardiac cine imaging (14, 15), first-pass cardiac perfusion (16), 3D late gadolinium enhancement (LGE) imaging (17), 3D whole-heart coronary MR angiography (CMRA), and more recently for 4D and 5D free-running CMRA (18–21), among many others. We briefly review some of those techniques in the next paragraphs.

#### Cardiac Cine Imaging

Cardiac cine MRI with CS reconstruction has demonstrated accurate estimation of cardiac function in a single-breath-hold (22). The study enrolled 81 patients with different cardiac conditions who were imaged using 2D cine acquisition, under three heart beats per slice, with high spatial (1.7 × 1.7 mm^2^) and temporal resolution (41 ms). A non-linear iterative SENSE-type reconstruction was performed with spatio-temporal regularization using redundant Haar wavelets. The reconstruction was performed inline in ~3 min for a stack of eight continuous short-axis image. CS reconstruction led to slightly worse image quality compared to conventional PI cardiac cine. A similar acquisition/reconstruction framework was performed on 100 patients referred for CMR in Vermersch et al. (23). Free-breathing 2D motion-corrected cine CMR has been also studied in Usman et al. (14). Acquisition was performed on five healthy subjects using a golden radial pseudo-random sampling and non-rigid respiratory motion-corrected reconstruction with CS temporal regularization was performed offline (reconstruction time ~2–2.5 h).

A 3D cardiac cine acquisition with CS reconstruction has been proposed to image the left ventricle in a single breath-hold (15). Ten healthy subjects and three patients were imaged at 1.9 × 1.9 × 2.5 mm^3^ spatial and 42–48 ms temporal resolution in ~19 s using a Cartesian spiral phyllotaxis sampling (24). Reconstruction times were ~4 min employing a soft-gated iterative SENSE reconstruction with spatial and temporal redundant Haar wavelet transforms. Free-breathing 3D cardiac cine has also been proposed to alleviate the requirement of breath-holding in Usman et al. (25). Whole-heart cardiac cine images were acquired in eight healthy subjects and three patients in ~4–5 min using an accelerated 3D free-running sequence with 2 mm^3^ isotropic resolution and ~31–70 ms temporal resolution. A CS-SENSE reconstruction with total variation regularization and translational respiratory motion correction was performed offline in ~2.5 h.

#### 3D Late Gadolinium Enhancement Imaging

CS has been employed to increase the spatial resolution and accelerate scan time of LGE imaging for myocardial scar and fibrosis visualization. Kamesh Iyer et al. (17) proposed a CS technique for rapid 3D LGE imaging for visualization of ablation-induced scar in the left atrium wall in patients with a history of atrial fibrillation and ablation therapy. 3D LGE data was acquired fully sampled on 8 patients and retrospectively undersampled using a variable density sampling with a 3.5-fold acceleration at a resolution of 1.25 × 1.25 × 2.5 mm^3^ (acquisition time of ~10–15 min). CS reconstruction was performed offline after coil compression (four virtual channels reconstructed) using an efficient Split Bregman optimization (26) for fast reconstruction (~8 s for 44 slices) with 3D total variation regularization. The Split Bregman method has shown to be an efficient solver for many regularized inverse problems with good convergence properties and fast minimization (26).

Basha et al. (27) proposed a patch-based CS technique (“LOST,” see next section) to acquire and reconstruct isotropic spatial resolution 1.4 × 1.4 × 1.4 mm^3^ 3D LGE data in 270 patients referred for myocardial viability assessment, using a pseudo random k-space undersampling pattern (28) with up to 5-fold accelerated acquisition (~4 min total acquisition time). LOST reconstruction was performed inline (via CPU cluster) in ~ 1 h.

#### Whole-Heart CMRA

Forman et al. proposed a free-breathing (29) and multi-breath-hold (28) Cartesian spiral phyllotaxis (6.5-fold) acquisition combined with an inline multi-coil SENSE reconstruction and 3D total variation regularization to reconstruct high-resolution (~1 mm^3^ isotropic) CMRA images in ~52 s. Accelerated non-rigid motion-compensated isotropic (1.2 mm^3^, 3-fold acceleration) 3D CMRA was also performed in ~5 min using 3D total variation regularization (reconstruction time ~44 min) and variable density Cartesian acquisition (30). Haar wavelets combined with an efficient FISTA optimization were used for whole-heart navigator-gated CMRA imaging at 3T, employing a Cartesian spiral phyllotaxis sampling at 9-fold acceleration (effective scan time of ~3 min 45 s at a resolution of 1.3 × 1.3 × 1.2 mm^3^) (31). A similar optimization was employed at 1.5T to reconstruct CMRA images with an isotropic resolution of 0.8 mm^3^ (32). CS techniques based on discrete wavelet transform were also implemented on GPU to bring whole-heart CMRA image reconstruction to <4 s (33).

XD-GRASP (34) and its extensions have been proposed to enable free-breathing whole-heart motion-resolved 5D [(*x*−*y*−*z*) spatial dimensions + respiratory and cardiac phases] CMRA in a single continuous acquisition by exploiting temporal total variation along the cardiac and respiratory dimensions (35–37). In Feng et al. (35), image acquisition was performed with a continuous 3D golden-angle pattern at isotropic 1.15 mm^3^ resolution and ~40–50 ms temporal resolution (acquisition time ~14 min). A conjugate gradient optimization was used to reach offline reconstruction times of ~6 h 48 min. Similar approaches were also proposed for time-resolved, cardiac-resolved, high-resolution flow imaging [XD flow (38)].

#### Drawbacks of CS for CMR

Although CS has shown noticeable success in CMR, as reflected by the many applications and recent integration into routine clinical scanners, there remains major drawbacks which may impede its full potential. Firstly, the non-linear nature of the optimization presents a barrier for fast reconstruction time, although notable improvement has been made on the maturation of the algorithms and the move toward GPU implementations to greatly reduced computational times. Another relevant weakness of CS-based reconstruction is the need for tuning regularization parameters that heavily depend on the type of image, sampling trajectories, sparsifying transform, acceleration factor, etc. Finally, while choosing the appropriate transformation basis Φ can contribute to an efficient sparse representation, the robustness of the reconstruction will heavily depend on this specific operator.

### Low-Rank-Based Approaches for CMR Imaging

Another model closely related to sparsity is the notion of low-rank matrices. Low-rank image reconstruction takes advantage of the fact that MR images have inherently a high degree of correlation (e.g., dynamically or locally on a patch scale) and thus can be represented by a union of low-dimensional subspaces. We provide below an overview of some reconstruction techniques incorporating low-rank models employed for CMR imaging.

Globally low-rank (GLR) reconstructions, exploiting low-rankness on the entire image series, have been exploited in many cardiac applications such as dynamic cine MRI (39–41), real-time CMR (42), cardiac perfusion (43), or simultaneous multislice CMR fingerprinting (44). GLR reconstruction techniques are particularly suited for image series that exhibit strong correlation over time. A Casorati matrix is usually formed from the undersampled image sequence, and the missing *k-t* samples are then estimated using low-rank matrix completion (41, 45, 46). Low-rank reconstruction has been combined with CS-based techniques to further improve image quality, particularly for high acceleration factors. Low-rank plus sparse (*L*+*S*) matrix decomposition, which separates the temporally correlated background (*L*) from the dynamic information (*S*), has been proposed for dynamic imaging (cardiac cine, cardiac perfusion, and time-resolved angiography) (43, 47). The recently proposed *multitasking* framework has extended global low-rank reconstruction to deal with multiple overlapping dynamics such as T1/T2 recovery and cardiac and respiratory motions, through tensor decomposition (48, 49).

Locally low-rank (LLR) regularization techniques have also been proposed for CMR reconstruction to further reduce spatial blurring often associated with the GLR techniques (50). In essence, LLR reconstruction techniques exploit low-rankness structure of an image series on local regions (i.e., patch), and have been efficiently used for dynamic CMR imaging (51, 52), high-resolution dynamic myocardial T1 mapping (53) and 5D flow (18).

**Figure 3:**
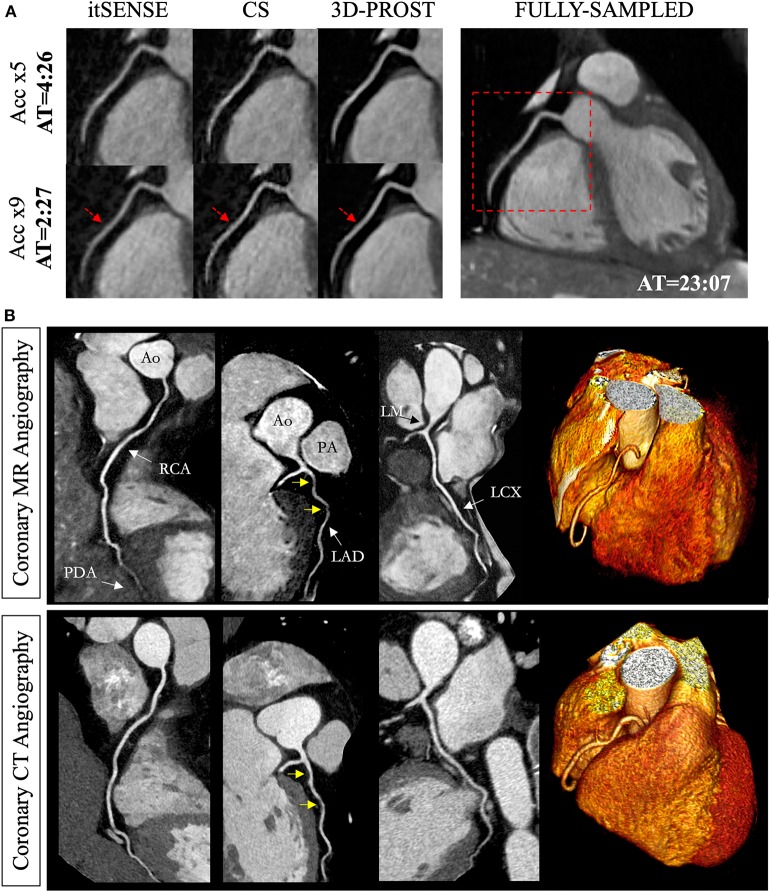
Reconstruction comparisons for coronary MR angiography. **(A)** Example of reformatted images of the right coronary artery from three healthy subjects acquired at 1.2 mm^3^ isotropic resolution with a fully sampled whole-heart coronary MR angiography sequence, and with 2 undersampled acquisitions (5- and 9-fold acceleration with variable density sampling), reconstructed using iterative SENSE (itSENSE), wavelet-based compressed sensing reconstruction (CS) and a 3D patch-based approach [3D-PROST (56)]. 3D-PROST provides higher image quality and sharpness (red arrows) than itSENSE and CS for both acceleration factors, achieving similar image quality to the fully sampled reference. Acquisition times (AT) are expressed as min:s. **(B)** Non-contrast whole-heart sub-millimeter isotropic CMRA images of 53-year-old female patient acquired in 10 min 7 s (5-fold undersampling) and reconstructed with 3D-PROST (56) and non-rigid motion correction (57). Visual comparison with contrast-enhanced Computed Tomography angiography (bottom row) shows good agreement and delineation of the coronary arteries with the free-breathing 3D patch-based motion corrected CMRA framework.

More recently, patch-based image reconstructions exploiting local (i.e., within a patch) and non-local (i.e., between similar patches) similarities and low-rank matrix representations have been employed for CMR image reconstruction, leading to even sparser representations. In those techniques (a.k.a. LOST and PROST, Figure 2) the similarity of 2D/3D image patches have been exploited through block-matching and low-rank decomposition. These techniques have shown to reconstruct highly undersampled LGE (27, 54) and CMRA images with improved image quality compared to CS-based techniques (55, 56) (Figure 3). Accelerated free-breathing CMRA in concert with 3D-PROST reconstruction enables isotropic sub-millimeter (0.9 mm^3^) whole-heart visualization of the coronary vasculature, including small distal segments, in ~5–7 min acquisition time and ~3 min reconstruction time (Figure 3). Based on a similar idea, patch-based reconstruction has been used for the reconstruction of undersampled 2D cine MR images by extending the patch search to the cardiac temporal dimension (58). The technique has been also extended to multi-contrast CMR reconstruction through high-order tensor decomposition (59) and demonstrated for highly accelerated simultaneous 3D myocardial T1/T2 mapping and cine imaging (60), and 3D whole-heart myocardial T2 mapping (61).

### Dictionary Learning-Based Approached for CMR Imaging

Dictionary learning based CS techniques (also referred as data-driven techniques) have been also proposed for CMR reconstruction. As opposed to conventional CS techniques, where sparse transforms or fixed dictionaries are known a priori, blind compressed sensing (BCS) techniques adaptively learn the sparse representation and dictionaries from the acquired undersampled data itself. These reconstruction techniques have the advantage to be highly adaptive to the image content at hand by learning dictionaries specific to the acquired data and without the need for training data. BCS has shown to outperform conventional CS approaches in several CMR applications such as cardiac cine MRI (62, 63) and contrast enhanced dynamic MRI (64).

Both dictionary learning and CS models can be leveraged to further increase acceleration factors. In Caballero et al. (62), a dictionary learning technique was combined with CS to speed up dynamic CMR imaging (~8- to 16-fold acceleration). An optimal dictionary is learnt directly from undersampled data online, through processing of spatio-temporal 3D patches, and is used to fill the missing k-space lines. The algorithm was tested on 10 healthy subjects by retrospectively undersampling fully sampled dynamic CMR data. Enforcing temporal gradients with an additional constraint allows to reach higher undersampling factors and accelerate the convergence rate, while consistently showing improvement over non-dictionary-based CS techniques.

Those approaches, however, come at the cost of highly non-convex optimizations, which make theoretical analyses and convergence guarantees very hard, while being often associated with high computational burden and long reconstruction times.

## Deep Learning for CMR Reconstruction

Despite the high promise of CS approaches, robustness of the reconstruction will heavily depend on the choice of the sparsifying transform which may be incapable of capturing the complex structure of CMR images. This may lead to images that look overly smooth or unnatural when too high acceleration factors are considered. A further major drawback is the long computational time usually required with iterative reconstruction algorithms and the need for parameters tuning. An inaccurate choice of reconstruction parameters leads either to over-smoothing or to images with remaining undersampling artifacts. Taking encouragement from early success in the use of DL in image classification and computer vision, several DL-based MRI reconstruction approaches have been recently proposed to learn models that better describe the reconstruction process and to shift the required optimization effort to an offline training stage, performed beforehand. In other words, rather than performing a reconstruction procedure to compute an appropriate transform between raw data and images for each new data set, DL reconstruction techniques propose to learn the parameters of that reconstruction procedure in advance, so that it can be applied to all new undersampled data as a simple operation. When using an analytical approach to solve Equation (3) for MR image reconstruction, the applied regularization operator is explicitly described, and the optimization approach is carefully chosen. Generally, the more sophisticated the modeling adopted in reconstruction, the more demanding the optimization process. The aim in DL-based MRI reconstruction, is to replace this optimization with a convenient function *f*_ϕ_(·) which is expressed as a DNN with parameters ϕ. Thus, a computationally efficient direct mapping from the acquired data *s* to the reconstructed image ρ can be obtained as a result of the neural network's training procedure. Training of a neural network implies changing its weights to optimize the network's output. This is performed by applying an optimization algorithm on a function measuring the difference of the outputs with respect to a target dataset, referred as loss function. Once these weights are learned, a network can be utilized to reconstruct new, unobserved data, and therefore learn to generalize. We will further discuss the training procedure in the section Training Procedure for DL-Based MRI Reconstruction. The main advantage of DL-based reconstruction techniques, with respect to conventional analytical reconstruction techniques, lies in the capability of a DNN to utilize the prior information learnt from the great number of routinely performed MRI exams, to help the reconstruction process. However, due to the problem's high dimensionality, a large dataset of raw k-space data *s* and target MRI images ρ need to be available to avoid over-fitting in the learning process. Collection of large MRI datasets can be challenging and proposed techniques for MRI reconstruction usually depend on the use of data-augmentation techniques, which is discussed in the section Data Availability for CMR Reconstruction. Given these preliminary remarks, a fundamental question may arise: Under which conditions would we expect DL approaches to outperform CS approaches in terms of reconstruction accuracy in CMR imaging (computational considerations aside)? In this section, we do not aim to provide a definitive answer to this question. Our objective is to provide the reader with a critical approach in reviewing the literature, to be used as guidance in solving their DL-based CMR reconstruction problems. DNN architectures and neural network training procedures will be described first for generic MRI reconstruction, followed by a review of the approaches that have been designed for cardiac applications.

### Neural Networks Architectures for DL-Based MRI Reconstruction

Careful selection and design of the neural network architecture is fundamental to solve the MRI reconstruction problem at hand, since the architecture's design controls the set of available functions *f*_ϕ_(·) that are investigated during the learning process. A *Neural Network* is composed of an input layer, followed by hidden layers that transform the data in a new representation; and it ends with an output layer that generates the neural network's prediction. Each layer is composed of multiple neuron units. The output of the neurons in each layer is given by the weighted sum of the input neurons, followed by a non-linear function termed *Activation Function*. A series of fully connected layers and activation functions is referred to as fully-connected neural network. The major advantage of fully connected networks is that they are “structure agnostic,” which means that no special assumptions need to be made about the network's input. In the following subsections we briefly discussed neural networks architectures that have been proposed to enable MR image reconstruction.

### Convolutional Neural Networks

Convolutional neural networks (CNN) (65) differ from fully-connected neural networks by the application of convolutions to each layer. As multiple convolution kernels are applied, several feature maps are defining a novel image characterization. In CNNs, there are usually less parameters with respect to fully-connected neural networks, since the kernel's weights are fixed as they move across the input image. The reduction in number of parameters simplifies the network's optimization problem. CNNs have been shown to learn interesting features from medical images and to be particularly appropriate to capture their multiscale structure. The use of residual blocks (66) also plays a fundamental role in training DNNs. Instead of learning a complete mapping function between consecutive layers; by adding skip connections between two or more layers, it is possible to learn the residual from the input to the output of a residual block or to the output of the whole neural network. The use of skip connection has been shown to be particularly well-suited to learn image features, such as edges or noise-like artifacts (66).

### Encoder-Decoder CNN

While for conventional CNNs feature map dimensions are fixed, for encoder-decoder CNNs the feature maps are gradually downsampled at each layer down to a convolution with a kernel of size 1 × 1, and then upsampled to the output's size. The first half of the network, the encoder part, learns a representation in a smaller manifold of the input image, and is then given as input to the decoder part of the network to obtain an image with the most meaningful features. Since the encoder part of the network compresses the feature maps' spatial information, a loss of details in the output can be encountered using an encoding-decoding network (67). This issue can be overcome by inserting symmetric skip connections, therefore preserving the important details that are present in the input image. An encoder-decoder network with skip connections is commonly referred to as U-Net network (67).

### Variational Neural Network

In the conventional CNN architectures described above, the input data is convolved with a set of filter kernels which are usually followed by a simple, non-learnable, activation function, e.g., rectified linear unit (ReLU). In a variational neural network (VNN), the regularization term *R* in Equation (3) is defined as a field of experts model (68):

(4) $$\begin{array}{l} R\left(\rho \right)=\overset{FK}{\underset{k=1}{\sum }}\langle \Psi_{k}\left(\chi_{k}\rho \right),1\rangle \end{array}$$

Where *R* is a linear operator that models convolutions of the image ρ with *FK* filter kernels $\chi_{k}\in R^{v\times v}$ of size *v*, and learnable non-linear activation function ψ_*k*_. In the fields of experts model (68), the convolutional kernels and the parameters of the non-linear activation functions are learned from the data. In contrast to other techniques that make use of ReLU, the parametrizable activation functions ψ_*k*_, used in Equation (4), are defined as a weighted combination of *AF* Gaussian radial basis functions. In a VNN architecture, the learning power is therefore shifted from the sole learning of the filter kernels to the learning of both kernels and non-linear activation functions.

### Training Procedure for DL-Based MRI Reconstruction

In the previous section, generic DNN architecture blocks have been described for solving MRI reconstruction problems. The choice of the architecture structure and of its constitutive elements determines a set of learnable functions, but it is during the training phase that the set of optimal functions for the given reconstruction task is determined. In general, the training procedure can be designed in a supervised or unsupervised fashion. Supervised methods are mostly used for MRI reconstruction, while unsupervised methods are an active topic of ongoing investigation. Therefore, for the rest of this section, we will focus on supervised approaches. In order to learn the network's parameters for the reconstruction procedure at hand, an optimization problem that minimizes a cost function needs to be defined. The training loss function can be defined as:

(5) $$\begin{array}{l} C\left(\phi \right)=\frac{1}{2B}\overset{B}{\underset{b=1}{\sum }}{\left\|\rho_{b}^{\Upsilon }\left(\phi \right)-\rho_{b}^{target}\right\|}_{2}^{2} \end{array}$$

Where ϕ are all the trainable parameters of the reconstruction network. Υ is the total number of layers in the network, corresponding to the network's gradient steps υ = 1, …, Υ. *b* is the current training output image. *B* is a randomly selected subset of the complete set of training data, referred as data batch. To solve the non-convex optimization problem in Equation (5), a variant of gradient descent, e.g., stochastic gradient descent or the ADAM optimizer are often used (69). The necessary computation of the gradient with respect to network parameters ϕ can be computed via backpropagation (70):

(6) $$\begin{array}{l} \frac{\delta C\left(\phi \right)}{\delta \phi^{\upsilon }}=\frac{\delta \rho^{\upsilon +1}}{\delta \phi^{\upsilon }}\cdot \frac{\delta \rho^{\upsilon +2}}{\delta \rho^{\upsilon +1}}\ldots \cdot \frac{\delta \rho^{\Upsilon }}{\delta \rho^{\Upsilon -1}}\cdot \frac{\delta C\left(\phi \right)}{\delta \rho^{\Upsilon }} \end{array}$$

These optimization algorithms require the tuning of hyper-parameters, such as strength of regularization or learning rate decay. The choice of the loss function is also crucial for a successful outcome of the training procedure. Because the reconstruction problem is usually formulated as a regression problem, the mean squared error is conventionally utilized as a cost function. Other popular choices are the *l*_1_ norm of the difference and the structural similarity index. Research on generative adversarial networks (71, 72) and learned content loss functions are currently in progress. Once the optimal parameters ϕ are learned, the reconstructed image ρ can then be estimated from the observed k-space data *s* by simply computing ρ = *f*_ϕ_(*s*) using the trained network. This efficient functional relationship is a major advantage of neural networks over conventional CS techniques that may require complex inference procedures (73).

### Data Availability for DL-Based CMR Reconstruction

The inference step between input and output of the reconstruction model is highly dependent on the set of input k-space data and of reference images seen during training. This requires the availability of a large set of fully sampled multi-coil k-space data. Undersampled data can be obtained by retrospectively removing k-space data entries according to a sampling trajectory in the forward operator E. This data can be used as input for the reconstruction network during training. The lack of freely accessible databases of fully sampled multi-channel raw k-space data, is an open issue for DL-based CMR reconstruction. In addition, since the dataset used to train a certain model becomes an essential component that defines its performance, it is difficult to compare different approaches if the training data is not publicly available. Even if initiatives for release of annotated CMR images are growing (e.g., UK Biobank), very limited public or institutional k-space CMR raw data have been provided to the research community. Moreover, large data bases of annotated CMR images, such us UK Biobank, are limited to specific type of exams. The DL reconstruction techniques presented in the following section are therefore mostly applied to retrospectively simulated k-space data and are restricted to specific MRI sequences (e.g., cardiac cine MRI).

### Neural Networks Architectures for DL-Based CMR Reconstruction

In this section, we review representative approaches proposed in the literature for MRI image reconstruction with a focus on CMR applications. The different approaches are summarized in Table 1.

**Table 1:** Summary of methods that, to the best of our knowledge, have used a deep-learning-based approach for CMR reconstruction and which have been referred to in this article.

**References**	**Application**	**Method/Network architecture**	**Training/Validation data**
Hauptmann et al. (74)	Cine MRI	3D U-net applied in post-processing to reduce streaking artifacts	Single-coil retrospective/Single-coil prospective
Kofler et al. (75)	Cine MRI	2D U-net applied to the spatio-temporal domain in post-processing	Single-coil retrospective/Single-coil prospective
Schlemper et al. (76)	Cine MRI	End-to-end cascade of CNN regularization blocks and data-consistency blocks	Single-coil retrospective/Single-coil retrospective
Fuin et al. (77)	CMRA	End-to-end cascade of Multi-Scale VNN regularization blocks with data-consistency operators	Multi-coil retrospective/Multi-coil prospective
Biswas et al. (78)	Cine MRI	End-to-end cascade of CNN operators, an analytically defined SToRM prior, and conjugate gradient data consistency steps	Multi-coil retrospective/Multi-coil retrospective
Qin et al. (79)	Cine MRI	End-to-end cascade of recurrent CNN regularization blocks and data-consistency blocks	Single-coil retrospective/Single-coil retrospective
Akçakaya (80)	Myocardial T1 mapping	CNN for k-space interpolation	Scan-specific Autocalibrating Signal data
Wang et al. (81)	Cine MRI	A first CNN for k-space interpolation followed by a concatenated CNN network architecture for image dealiazing	Single-coil retrospective/Single-coil retrospective

#### Encoder-Decoder CNN for Image Dealiazing

**Figure 4:**
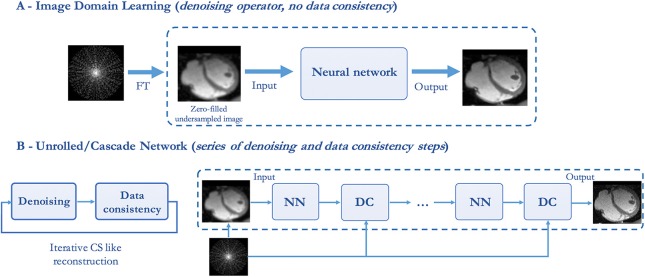
Illustration of two types of deep learning-based image reconstruction networks. **(A)** Image domain networks and **(B)** End-to-end unrolled networks, where NN denotes a CNN or VNN denoising operator and DC denotes the data consistency layer.

U-net type of networks that perform an end-to-end mapping in image space have been successfully employed in many MRI post-processing applications (e.g., image segmentation) showing promising results. In the field of image recovery from undersampled k-space data, U-net architectures have been used by several groups to reduce noise-like image artifacts in post processing (see Figure 4A).

**Figure 5:**
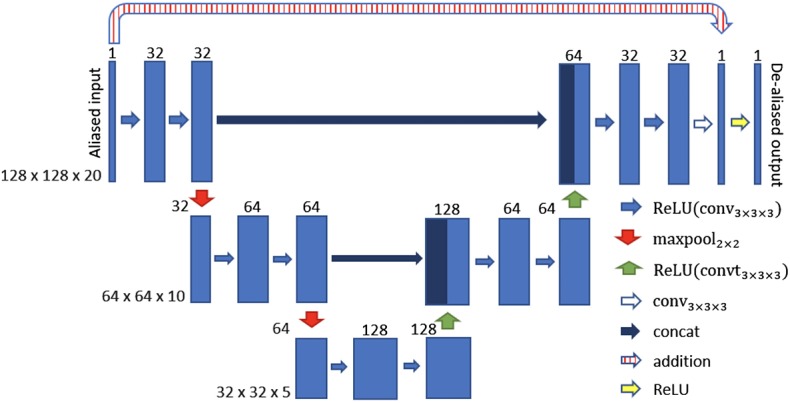
3D U-net architecture for cine MRI spatio-temporal de-aliasing. Reconstructions from undersampled cine MRI data are given as an input. The numbers on top of the blue bars denote the number of channels for each layer. The resolution for each multilevel decomposition is shown in gray on the left. Each convolutional layer is equipped with a rectified linear unit (ReLU) as non-linear activation function. The residual U-net contains a skip connection at each scale between encoder and decoder path (concat and/or addition).

**Figure 6:**
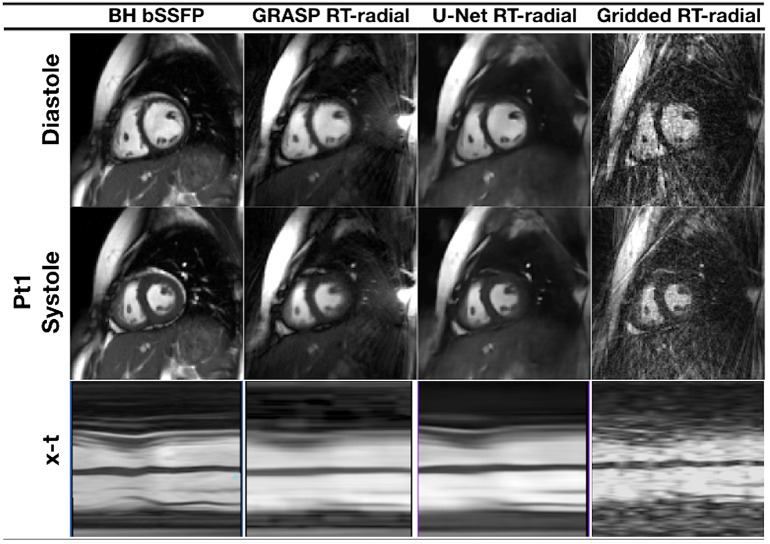
Cine MRI images for one representative patient with congenital heart disease, acquired with prospective undersampling of 13-fold. Reconstructed images are presented in peak systole and peak diastole for a reference breath-held balanced steady-state free precession sequence (BH-bSSFP, first column), the real-time radial sequence reconstructed with GRASP (82) (second column) and the residual 3D U-net (third column), as proposed in Ronneberger et al. (67). Images reconstructed with GRASP and the proposed residual 3D U-Net show spatial and temporal blurring, that could be a result of undersampling and incomplete motion correction.

In Hauptmann et al. (74), a 3D residual U-net have been employed to reduce undersampling artifacts for 2D golden-angle radial cardiac cine MRI. This residual U-Net contains a contracting multi-scale decomposition path and a symmetric expanding path with skip connections at each scale (see Figure 5). The 3D-convolutions are trained on entire image sequences (*x* − *y* − *t*) to enforce temporal consistency between cardiac frames. This technique demonstrated robustness with respect to the flickering artifacts that would be present if 2D convolutions were separately applied to each frame. The proposed U-net architecture was trained from 13-fold retrospectively undersampled images using a simulated tiny golden angle radial trajectory. These images were obtained from Cartesian breath-hold (BH) bSSFP cine acquisitions of 250 patients with congenital heart disease (CHD). The trained 3D U-net was then applied to real-time 13-fold accelerated tiny golden angle 2D radial bSSFP data acquired under free-breathing in 10 previously unseen patients with CHD. The radial bSSFP data were recovered with the proposed 3D U-net and reconstructed with CS for image quality and computational time comparisons. Ventricular volume measurements for 10–15 contiguous slices, obtained using both the CS reconstructed images and 3D U-net, were compared to a reference Cartesian fully sampled BH-bSSFP cardiac cine data. The overall reconstruction time with the residual 3D U-net implemented on graphics processing unit (GPU) was five times faster than conventional CS techniques implemented on CPU (74). Moreover, the overall image quality of the ventricular volume measurements from the 3D U-net recovered images were superior than the CS reconstructions (Figure 6). In this study, the validation data was acquired during free-breathing, while the training data was obtained during a breath-hold; the effects of cardiac and respiratory motions were therefore not taken into consideration.

**Figure 7:**
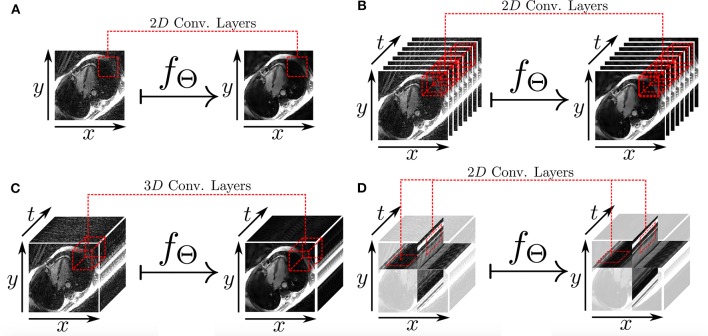
Different 2D and 3D deep learning-based approaches for radial undersampling artifacts reduction (post-processing) presented in Kofler et al. (75). **(A)** 2D U-net for frame-to-frame mapping. **(B)** 2D U-net for sequence-to-sequence mapping with cardiac phases aligned along the channel dimension. **(C)** 3D U-net for sequence-to-sequence mapping with 3D convolutional kernels. **(D)** 2D U-net for recovery of two-dimensional spatio-temporal images.

The work presented in Hauptmann et al. (74) demonstrates that 3D CNNs can be employed to map entire undersampled 2D sequences to the corresponding fully-sampled 2D cardiac cine sequences. However, employing 3D convolutional layers requires a higher number of parameters and thus increases the amount of data needed to efficiently train a network and prevent overfitting. In Kofler et al. (75), the authors proposed a technique to recover undersampled 2D golden-angle radial cine CMR by training a modified 2D U-net on the 2D spatio-temporal domain (*x* − *t*) extracted from the image sequences (Figure 7). This study suggests that the learning process can be improved by training the network on 2D *x* − *t* images extracted from the spatio-temporal domain of the cardiac cine sequence. This technique obtained similar results with respect to the 3D U-Net (74) by training the network on a substantially smaller training data set and also proved to be robust with respect to rotations in image space.

The main limitation of the approaches presented in this section, as for all DL techniques applied in post-processing, is that the actual validation data consists of coil-combined magnitude images, instead of multi-coil complex k-space data. Therefore, these approaches do not learn a full reconstruction procedure that accounts for consistency with respect to the acquired k-space data (see Figure 4), but also do not take advantage of the full benefits of coil sensitivity encoding underlying parallel imaging.

#### Unrolled Convolutional Neural Networks

In this section, we describe how a DNN can be guided to learn operations that are similar to those performed in conventional iterative CS reconstruction, therefore bridging the gap with conventional iterative techniques. Incorporating domain expertise in a DNN framework can in fact facilitate the learning procedure of the model and result in better estimates of the MR images. For CS-based variable splitting techniques, the optimization problem in Equation (3) is usually solved using an alternating algorithm, iterating between a regularization stage and a data consistency stage. Instead of explicitly defining the regularization term, several DL techniques have been proposed to directly learn the regularization term by using CNNs. These techniques, such as Deep-ADMM net (83), VNN (84), or CascadeNet (76), represent a DL framework of an unrolled version of the iterative constrained reconstruction where the network parameters are trained in order to reconstruct the MR images directly from the undersampled k-space data as an input (see Figure 4B).

**Figure 8:**
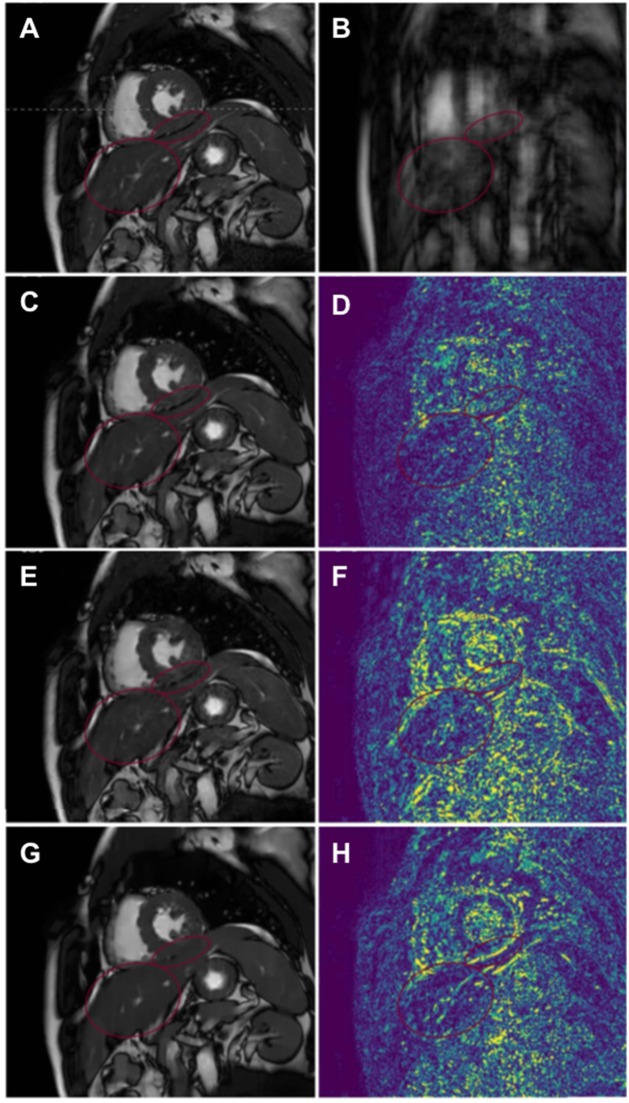
Comparison of reconstructed 2D cardiac cine MR image sequences employing Dictionary Learning with Temporal Gradient (DLTG) (62) and CascadeNet (CNN-S) (76), from one representative healthy subject with retrospectively undersampling. **(A)** Ground truth fully-sampled cine MR image, **(B)** 9x retrospectively undersampled acquisition, **(C,D)** CascadeNet reconstruction with data sharing and its error map, **(E,F)** CascadeNet reconstruction without data sharing (CNN) and its error map, **(G,H)** DLTG reconstruction and its error map. Red ellipses highlight the anatomy that was reconstructed better by CNN than DLTG.

In particular, Schlemper et al. (76) proposed a framework for the reconstruction of 2D cardiac cine MR images from highly undersampled data using a cascade of CNNs, termed CascadeNet. Since a simple CNN is not efficient in learning the regularization operator iteratively; the authors proposed to concatenate a new CNN on the output of the previous CNN to create a DNN that iterates between CNN regularization operators and data consistency operators. The resulting network consists in convolutional layers, followed by ReLU, residual connections, and data consistency layers. The authors employed a hard-projection solution to enforce data consistency: for each stage of the unrolled model, if the k-space samples are initially unknown (non-acquired), then k-space values obtained from the FT of the previous layer's output are used. For the k-space entries that have been acquired, a linear combination between the estimated values from the previous layer and the original measurements is applied. Since the data consistency step has a simple expression, it is possible to treat it as a layer of a network and to specify the rules for forward and backward propagation for training. By defining the forward and back-backpropagation rules for the data consistency layer, all stages of the network can be trained in an end-to-end fashion, therefore building one deep network. The authors also demonstrated that spatio-temporal correlations can be efficiently learned by CNNs, combining 3D (*x* − *y* − *t*) convolutions and data sharing approaches. Assuming that for adjacent cardiac frames the difference in data content is relatively small, the neighboring k-space frames along the temporal-axis share similar information. The missing k-space samples for each time frame can then be approximated using the samples from the adjacent cardiac frames. The authors therefore extended the proposed network architecture adding data “sharing layers that take an input image and generate multiple data-shared images” (76). The obtained images are then concatenated along the channel-axis of the network and fed into the proposed cascading network. For separate reconstruction of 2D cardiac single frames, this technique was compared to Dictionary Learning MRI (85), for retrospective undersampling factors of 3- and 9-fold. For reconstruction of cardiac cine MRI, the technique was compared to state-of-the-art CS and low-rank approaches, such as dictionary learning with temporal gradient (62), *k-t* sparse and low-rank (kt-SLR) (46), and L+S matrix decomposition (43). The presented results demonstrated that the CascadeNet outperforms CS and low-rank approaches in terms of reconstruction error and perceptual quality, particularly for high undersampling rates (Figure 8). In addition, for 2D reconstruction, each image could be reconstructed in 23 ms, therefore enabling real-time applications, while for the reconstruction of cine MRI, an entire sequence was reconstructed within 10 s.

It is worth noting that in the experiments shown in Schlemper et al. (76), training and validation data were obtained by retrospectively undersampling single-coil data, thus further validations are required to understand the full potential of this technique for multi-coil prospective acquisitions. Other techniques have applied an unrolled end-to-end framework in the more realistic scenario of multi-channel coil complex MR data. For example, Hammernik et al. proposed a trainable formulation for undersampled MRI reconstruction (84), which embedded a PI and a CS reconstruction within a DL unrolled end-to-end framework. Undersampled k-space data and coil sensitivity maps are provided as input to this unrolled model for DL reconstruction, and high-quality MR images are obtained as an output in an end-to-end fashion. The regularization term of this network was implemented as a VNN, and the data consistency term was implemented as the *l*_2_ norm with respect to the acquired k-space data, as in Equation (3). The use of a VNN was first introduced for multi-coil complex-valued MRI reconstruction of 2D static images of the knee.

**Figure 9:**
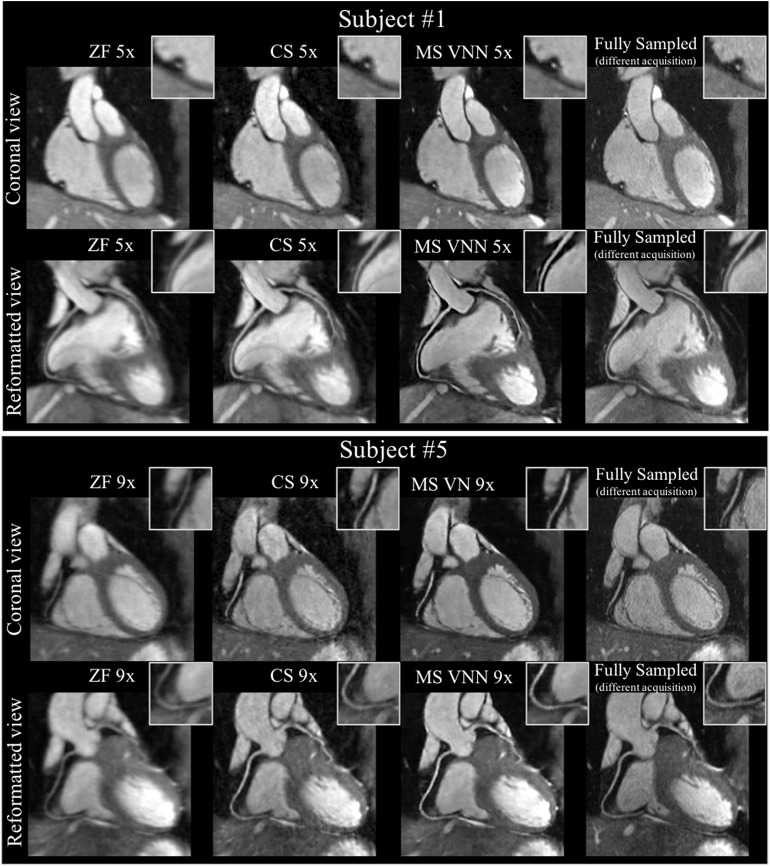
Coronary MR angiography images in coronal view and reformatted along the left (LAD) and right (RCA) coronary arteries, for one representative healthy subject. Acquisitions were performed with isotropic resolution 1.2 mm^3^ and 100% respiratory scan efficiency (no respiratory gating). Prospective undersampled acquisitions with acceleration factors 5x (first and second rows) and 9x (third and fourth rows) are shown. Images were reconstructed using zero-filling (ZF), a wavelet-based CS reconstruction (CS), and the Multi-Scale VNN (MS-VNN) reconstruction framework proposed in Fuin et al. (77). Corresponding (consecutively acquired) fully sampled acquisition are shown in the last column for comparison. Multi-scale VNN provides higher image quality than ZF and CS achieving similar image quality to the fully sampled scan. Reconstruction time was ~14 s with MS-VNN and ~5 min with wavelet-based CS.

Building on this work, Fuin et al. (77) extended the previously introduced VNN approach to enable fast reconstruction of undersampled motion-compensated free-breathing whole-heart 3D CMRA. A multi-scale VNN (MS-VNN) architecture was introduced in order to better capture the small caliber of the coronary arteries, as well as whole-heart structural features (*x* − *y* − *z*) in a 3D CMRA image. In order to increase the representation potential of the network, a wider network was implemented, using a multi-scale approach that can capture complementary and richer information at different resolutions. In addition, a training scheme suited for reconstruction of respiratory motion corrupted data was applied. The MS-VNN was trained on retrospectively undersampled (5- and 9-fold) translational motion corrected complex k-space data in an end-to-end fashion, in order to ensure that the effect of bulk, respiratory, and cardiac motion was identical in both output and target images during the training process. The MS-VNN reconstruction was then applied to newly acquired prospectively 5- and 9-fold undersampled data and compared to wavelet-based CS (12) reconstructions, as presented in Figure 9. MS-VNN outperformed the conventional CS in terms of quantitative right coronary artery sharpness and visible vessel length, with results comparable to the fully sampled scan. MS-VNN combined with 100% respiratory scan efficiency and variable density spiral-like Cartesian undersampling, allowed the acquisition of high-quality 1.2 mm^3^ isotropic CMRA images in a short and predictable scan time of ~2–4 min and their reconstruction in ~14 s.

Aggarwal et al. (86) introduced a similar network design, termed MoDL, where conventional CNNs are used for the implementation of the regularization term, but where all network stages share the same set of parameters. This unrolled technique with shared parameters, also applies a conjugate-gradient data consistency step instead of the simple gradient based approach utilized in Hammernik et al. (84). The use of a conjugate-gradient step within the network translates into improved results for a given number of iterations at the expense of a slightly longer run time. Another work from the same team combines DL MoDL reconstruction along with complementary analytical image regularization constraints to recover free-breathing cardiac cine MR images from highly undersampled multi-coil measurements (78). This framework alternates between a learned regularization of the image using CNN, an analytically defined SmooThness regularization on manifolds (SToRM) prior (87), and a conjugate gradient data consistency step. The method was tested on only two simulated datasets, but it promises to combine the benefits of CNNs with analytical image regularization priors, such as SToRM, which exploits subject-specific information including cardiac and respiratory patterns.

#### Unrolled Convolutional Recurrent Neural Networks

A recurrent neural network can be thought of as multiple copies of the same network stage, each passing a message to a successor stage. The stage of the recurrent network has a memory that stores the stage time states, and therefore it allows information to be reflected to the next time stage without overloading the system. Qin et al. (79) proposed a novel unrolled convolutional recurrent neural network architecture, termed CRNN-MRI, which reconstructs cine CMR images from highly undersampled k-space data. The proposed CRNN-MRI architecture utilize recurrent connections over each layer of an unrolled network with data consistency layers to reproduce the recurrence existing in the sequential steps of a reconstruction algorithm. Compared to independently learned CNN at each stage of an unrolled network (76), the iteration connections of the CRNN layers allow spatial information learned at a given iteration to be passed to the following iteration. Each stage of the network is therefore optimized depending on the resulting output but also depending on features from previous iterations that can memorize the learned feature and propagate them to the next stage. Secondly, at every stage of the network, the receptive field of a CNRR layer in the spatial domain increases, whereas for a conventional CNN it resets at each stage. Finally, since the network parameters are shared over iterations, the total number of parameters is greatly reduced in comparison to CNNs, potentially offering improved generalization properties. An additional limitation of CNNs is that they accept fixed-sized images as input and produce a fixed-sized image as output. Conversely, recurrent nets allow to operate over sequences of images: sequences in the input, the output, or in the most general case in both input and output. Exploiting this property of recurrent networks, the network architecture presented in Qin et al. (79) incorporates bidirectional recurrent convolutional layers that evolve over time to utilize the temporal correlations of the cardiac cine MRI. Consequently, the model architecture evolves in a recurrent manner over time and over steps/iterations. The CRNN-MRI network therefore comprises of bidirectional convolutional recurrent layers, residual connections and hard-projection data consistency layers [as in (76)]. The residual connections were added to address the potential problem of vanishing gradients during back-propagation. Training and validation data were produced by retrospective undersampling complex images obtained from single-coil data as in Schlemper et al. (76). The experimental results demonstrated that CRNN-MRI outperformed state-of-the-art CS-based dynamic MRI and low-rank reconstruction algorithms, such as *k-t* FOCUSS (88) and *k-t* SLR (46) for 9- and 16-fold retrospectively undersampled data. Additionally, CRNN-MRI demonstrated to outperform CascadeNet (76), that employs conventional CNNs in the regularization term.

#### DL Techniques for K-Space Based CMR Reconstruction

One of the most frequently used techniques for PI undersampled reconstruction in k-space is GRAPPA (89), which employs shift-invariant convolutions to recover/interpolate non-acquired k-space entries. The convolutional kernels, called autocalibrating signal (ACS), are estimated for each subject from either a fully sampled region at the k-space center or from a separate reference scan (autocalibrating signal or ACS). A CNNs based technique has been recently proposed to improve non-linear k-space interpolation for undersampled PI MRI reconstruction (80). Similar to existing approaches, such as non-linear GRAPPA (90), robust artificial-neural-networks for k-space interpolation (RAKI) (80) trains CNNs on ACS data with an *l*_2_ norm loss; and uses these for interpolating missing k-space samples from acquired ones. The RAKI network architecture was applied for the reconstruction of myocardial 2D T1 mapping data. Eleven images with different T1 weights were acquired in a single breath-hold using a Cartesian fully sampled bSSFP sequence. Experimental results were then performed on 4- and 5-fold retrospectively undersampled data and RAKI showed improved noise resilience with respect to non-regularized GRAPPA reconstruction. As RAKI is a scan-specific technique and does not require a training data base, it could in theory be applied for the reconstruction of CMR data for which a fully sampled reference acquisition scan cannot be performed, as for example in perfusion or real-time CMR. However, being scan-specific, this approach also comes with downsides, such as high computational burden, computationally expensive training of a neural network for each scan, and the requirement for additional calibration data.

Recently, a technique that combines DL for k-space interpolation and image dealiazing for retrospectively undersampled 2D cardiac cine MRI has been proposed (81). This approach consists of a first frequency domain network architecture for k-space data interpolation followed by a concatenated image domain network architecture for image dealiazing. Both networks consist of concatenated CNN and ReLU layers, followed by a data consistency layer. The first and second networks are connected by a Fourier inversion and only one pass through the network is performed. Additionally, the authors propose a multi-supervised network training technique to constrain the frequency domain information and spatial domain information at different levels.

## Discussion

During the last decades, several undersampled MR reconstruction techniques have been developed to speed up CMR acquisition. These techniques rely on acquiring less data than needed (in the Nyquist sense) and estimating the non-acquired data exploiting some sort of prior information about the images. PI and CS undersampling reconstruction techniques have revolutionized the field, enabling high scan time accelerations to become standard in clinical practice. Despite of its maturity and recent FDA approval for clinical use, some major technical issues associated with CS reconstruction for CMR remain, including high complexity of the algorithms and long reconstruction times, image degradation at high accelerations, and the need for parameters tuning. Therefore, recent AI-based scientific advances have emerged as solutions to transfer the complexity of the CMR reconstruction from the inline side to the offline training side. Unlike analytical techniques for which the reconstruction problem is explicitly defined into the optimization process, DL-based techniques employ large data sets to learn the key reconstruction parameters and priors during an up-front training procedure, providing a fast and efficient reconstruction that can be applied to all newly-acquired cardiac data.

### Strengths and Recent Advances in AI for CMR Reconstruction

The sudden resurgence and popularity of DL approaches for medical image reconstruction can be attributed to their ability to analyze high-dimensional datasets, the availability of computing power, algorithms, web-based storage information, and real-time reconstruction. Although the application of DL to CMR reconstruction is still at an early stage, promising cardiac applications (e.g., dynamic cine MRI or CMRA) have been proposed.

In particular, end-to-end unrolled neural networks models have shown great potential to obtain CMR images that are comparable, in terms of anatomical structure and features, to images obtained with conventional iterative techniques. For example, MS-VNN (77) has shown to obtain high quality static images for prospectively undersampled whole-heart 3D CMRA imaging. Cascade-Net (76) and CRNN-MRI (79), were specifically designed for dynamic imaging and have demonstrated to outperform conventional CS techniques for retrospectively undersampled 2D cardiac cine MRI. Fewer techniques exist for the use of DNN as a k-space estimation problem. This may be due to the non-uniform features of the k-space data (especially for non-Cartesian trajectories), which make it difficult to translate some of the DL techniques that have been developed for image processing of natural images to CMR reconstruction. However, techniques such as RAKI (80) are scan-specific and do not require a training database; and thus, could in theory be applied to cases for which a reference fully-sampled acquisition cannot be performed.

### Limitations and Pitfalls

Although DL-based reconstruction techniques for CMR are showing promising results, there are several remaining challenges that need to be addressed before enabling widespread clinical use.

#### Simulation and Lack of Clinical Validation

Most of the existing early DL-based techniques for CMR reconstruction are purely based on simulated data, using retrospective undersampling experiments on fully sampled datasets, and limited to single-coil MR acquisition model. Therefore, it remains to be seen how those techniques will work in a multi-coil setting with prospective undersampling, where additional factors can drastically disrupt the reconstruction and degrade the image quality (e.g., eddy current related effects due to gradient jump, blurring due to off resonant spins with spiral trajectories, more complex noise models, unknown coil sensitivity profiles, cardiac and respiratory motion) and intrinsically result in a reduction of the achievable acceleration factor. Furthermore, those different studies have been so far limited to healthy or small selected patient cohorts, which unfortunately limits their current clinical applicability and clinical impact in more complex scenarios. Further clinical validations are thus warranted to demonstrate the robustness of those techniques.

#### Generalization and Reconstruction Quality

A key strength of CMR is the ability to provide images with different contrast for a comprehensive assessment of the disease. Therefore, one open question regarding the applicability of DL-based reconstruction techniques, in practice, is generalization. The generalization potential and effectiveness of these reconstruction techniques should be further investigated in case of, for example, different imaging resolutions, pulse sequences, acquisition trajectories, magnetic fields strength, MR vendors or clinical sites. While it would be feasible to pre-train separate neural networks for different exams, the poor generalization performance of a DL model to different sequence settings, anatomy, physiology, or to unique pathologies, will limit its translation into clinical practice. On this account, there is still an open question that needs to be investigated: can we design a reconstruction network which accurately and precisely extract unique information from limited samples, while generalizing to different acquisition settings and pathologies?

#### Data Availability

Another major drawback of DL reconstruction approaches lies in the availability of a specific training data set. The approaches presented in the previous sections have been trained on small samples of hundreds of cases rather than millions, as it is often the case in DL for classification or computer vision. However, the training of reconstruction network still requires the availability of organized and specific data sets that will allow the model to generalize toward new, unseen, test data. Moreover, most of the models presented are developed for few specific cardiac sequences, such as cardiac cine MRI, for which large image datasets are available to researchers (e.g., UK Biobank).

#### Quality of the Training Set

In addition to its size, the quality, and composition of the training set is of utmost importance. Several sequences in CMR, e.g., sub-millimeter CMRA or real-time CMR, cannot be acquired with fully-sampled data due to resolution and time constraints. This hinders the application of supervised training approaches for such datasets, justifying the necessity for future research in scan-specific strategies or unsupervised training. We anticipate that future research could focus on the development of neural networks architectures designed to learn features from different cardiac modalities or different MR acquisitions from other organs, in an unsupervised manner, and the incorporation of more conventional regularizations into the networks. The selection of the cost function also has an influence on the network training and optimization, and it is therefore the topic of currently ongoing research. Research on generative adversarial networks and learned content loss functions are also under progress.

#### Motion Compensated Reconstruction

Additionally, the considerable respiratory- and cardiac-induced motion of the heart during the MR acquisition can significantly impair image quality by showing blurring and/or ghosting like artifacts. Multiple accelerated motion corrected reconstruction frameworks have been developed to simultaneously accelerate scan time and correct for motion during reconstruction. In conventional iterative reconstruction approaches, it is more straightforward to account for motion correction in the reconstruction, as a non-rigid motion model can be directly included in the encoding operator *E*. Some preliminary simulation work in DL reconstruction have tackled the problem of correcting motion-related artifacts in 2D cardiac cine images during reconstruction by adding an adversarial element to the network architecture (91). However, no DL reconstruction technique has yet explicitly modeled non-rigid motion directly in the reconstruction process. The efficient implementation of 3D non-rigid transformations in a DNN architecture could in fact prove to be challenging and research on the topic is currently in progress.

#### Workflow Integration

Finally, most of the DL techniques proposed for CMR reconstruction are implemented offline. Whilst this may be suitable for initial testing, the inline integration of those techniques will be key for their full adoption in clinical practice. Several frameworks, such as Gadgetron (92) or Yarra (https://yarra.rocks), have already been proposed for the easy integration of in-house reconstruction techniques into MR scanners; we expect them to play a key role for supporting DL-based reconstruction as well. Many clinical cardiac applications, such as real-time MR-guided cardiac interventions (93) will largely benefit from such inline real-time reconstruction.

## Author Contributions

AB, NF, RB, and CP devised and wrote the manuscript.

### Conflict of Interest

The authors declare that the research was conducted in the absence of any commercial or financial relationships that could be construed as a potential conflict of interest.
